# Atomic Surface Segregation and Structural Characterization of PdPt Bimetallic Nanoparticles

**DOI:** 10.3390/ma11101882

**Published:** 2018-10-02

**Authors:** Carlos A. Rodríguez-Proenza, Juan P. Palomares-Báez, Marco A. Chávez-Rojo, Amado F. García-Ruiz, Cristy L. Azanza-Ricardo, Alan Santoveña-Uribe, Gabriel Luna-Bárcenas, José L. Rodríguez-López, Rodrigo Esparza

**Affiliations:** 1 Posgrado en Ciencia e Ingeniería de Materiales, Centro de Física Aplicada y Tecnología Avanzada, Universidad Nacional Autónoma de México, Campus Juriquilla, Santiago de Querétaro 76230, Qro., Mexico; carlosalbertorodriguezproenza@gmail.com (C.A.R.-P.); alansantovenia@hotmail.com (A.S.-U.); 2 Facultad de Ciencias Químicas, Universidad Autónoma de Chihuahua, Circuito Universitario s/n, Campus II, Chihuahua 31125, Chih., Mexico; mchavezrojo@gmail.com; 3 UPIICSA-COFAA, Instituto Politécnico Nacional, Te 950, Col. Granjas-México, Iztacalco, México 08400, D. F., Mexico; amado.garcia@gmail.com; 4 Centro de Física Aplicada y Tecnología Avanzada, Universidad Nacional Autónoma de México, Boulevard Juriquilla 3001, Santiago de Querétaro 76230, Qro., Mexico; cristy_azanza@fata.unam.mx; 5 Centro de Investigación y Estudios Avanzados (CINVESTAV) del Instituto Politécnico Nacional (IPN), Unidad Querétaro, Fracc. Real de Juriquilla, Santiago de Querétaro 76230, Qro., Mexico; glunascf@yahoo.com; 6 Departamento de Materiales Avanzados, Instituto Potosino de Investigación Científica y Tecnológica, A.C., San Luis Potosí 78216, SLP, Mexico

**Keywords:** bimetallic nanoparticles, electron microscopy, molecular dynamics simulation, PdPt alloys, nanostructures

## Abstract

Bimetallic nanoparticles are of interest since they lead to many interesting electrical, chemical, catalytic, and optical properties. They are particularly important in the field of catalysis since they show superior catalytic properties than their monometallic counterparts. The structures of bimetallic nanoparticles depend mainly on the synthesis conditions and the miscibility of the two components. In this work, PdPt alloyed-bimetallic nanoparticles (NPs) were synthesized through the polyol method, and characterized using spherical aberration (Cs) corrected scanning transmission electron microscopy (STEM). High-angle annular dark-field (HAADF)-STEM images of bimetallic nanoparticles were obtained. The contrast of images shows that nanoparticles have an alloy structure with an average size of 8.2 nm. Together with the characterization of nanoparticles, a systematic molecular dynamics simulations study focused on the structural stability and atomic surface segregation trends in 923-atom PdPt alloyed-bimetallic NPs was carried out.

## 1. Introduction

Bimetallic nanoparticles are of wide interest since they show physical and chemical properties that are distinct from those of their monometallic counterparts, and they present a combination of the properties associated with the two metals and create synergistic effects, which signifies the whole being greater than the sum of its parts [1]. Bimetallic nanoparticles have emerged as a new class of catalysts mainly in energy issue, with potential applications in advanced energy conversion and storage (ECS) devices, including fuel cells, photo-electrochemical water splitting cells, solar cells, Li-ion batteries, and supercapacitors, among others [2].

It is known that platinum (Pt) is one of the best single metal catalysts and is used mainly in the chemical industry, the petrochemical industry, automobile exhaust purification, and fuel cells because of its excellent reactivity and stability [3]. Improving the activity and utilization efficiency of Pt catalysts is therefore the key issue in the development of relevant catalysis fields. Normally, there are two types of target products to reduce Pt loading: Pt monometallic nanoparticles (NPs) with open surface structure [4] and Pt-based bi- (or multi-) metallic NPs [5]. Meanwhile, synthesis of Pt-based bimetallic NPs, as an alternative strategy, has received considerable attention as well because of their enhanced catalytic activities as compared to conventional Pt monometallic NPs [6,7,8,9]. For example, Pt-Au bimetallic NPs exhibited higher electrocatalytic activity and durability for oxygen reduction reactions than Pt monometallic catalysts [6,7]. Moreover, the superior activities of both Pt-Ru and Pt-Rh bimetallic NPs were also ascertained in preferential oxidation of CO in hydrogen [8,9].

Among Pt-based nanocatalysts, Pt-Pd is one of the most attractive bimetallic systems due to its highly promising application as a catalyst in formic acid oxidation and fuel cells [10,11,12]. Furthermore, Pt-Pd nanocatalysts have been considered as preferred substitutes of Pt-Ru catalysts for low-temperature direct formic acid fuel cell (DFAFC) technology owing to the much cheaper price of Pd [12]. PdPt bimetallic NPs can be produced generally by either successive co-reduction of Pt and Pd species or a seed-mediated growth method [13].

As it is known, the structure of bimetallic nanoparticles mainly depends on the synthesis methods and the miscibility of the two components [14]. The experimentally synthesized Pt-Pd bimetallic NPs can be classified into three configurations: Pt-core/Pd-shell, Pd-core/Pt-shell, and Pt-Pd alloyed nanostructures [10,15,16]. It is well-known that the catalytic activity of these NPs strongly depends not only on the particle size but also the Pt/Pd ratio [17]. Therefore, the effects of core sizes, shell thicknesses, and alloy compositions are urgent topics that should be addressed for further tailored design of highly active Pt-Pd nanocatalysts. Furthermore, considering that catalysts are generally used at different temperatures, the structural and thermal stabilities of PdPt bimetallic NPs are also important issues that need to be clarified [18]. Although considerable experimental and theoretical studies have been dedicated to design, synthesis, characterization and performance evaluation of Pt-Pd bimetallic NPs [19], there is still lacking a full atomic-level understanding of structural and thermal stabilities of PdPt bimetallic NPs with different configurations and compositions.

In this work, PdPt alloyed-bimetallic NPs were synthesized through the polyol method, and characterized using spherical aberration (Cs) corrected scanning transmission electron microscopy (STEM). High-angle annular dark-field (HAADF)-STEM images of the bimetallic nanoparticles were obtained. The contrast of the images shows that NPs have an alloy structure with an average size of 8.2 nm. In addition, a systematic study on the structural and thermal stabilities and atomic surface segregation of cubo-octahedral Pt and Pd monometallic and PtPd alloyed-bimetallic NPs using the classical molecular dynamics simulations was performed. Additionally, the dynamics of thermally driven structural evolutions and the atomic diffusive behaviors of the NPs were explored.

## 2. Materials and Methods

### 2.1. Synthesis of Bimetallic Nanoparticles

PdPt nanoparticles were prepared by oil-bath heating in ethylene glycol (EG, Sigma-Aldrich, St. Louis, MO, USA) using a one-step reduction method. Briefly, 3 mL of EG were preheated until the temperature was stabilized at 160 °C in a 3-neck round bottom flask. Over the stirred EG, 1.5 mL of 50 mM chloroplatinic acid hexahydrate (H_2_PtCl_6_·6H_2_O, Sigma-Aldrich, St. Louis, MO, USA) in EG, 1.5 mL of 50 mM potassium tetrachloropalladate (K_2_PdCl_4_, Sigma-Aldrich, St. Louis, MO, USA) in EG and 6 mL of 100 mM polyvinylpyrrolidone (PVP, Sigma-Aldrich, St. Louis, MO, USA) in EG were added slowly in aliquots of 200 µL (H_2_PtCl_6_·6H_2_O and K_2_PdCl_4_) and 400 µL (PVP), respectively, each 2.5 min until the volume of the precursor and surfactant was completed, then the mixture was kept on stirring at 160 °C for 2 h. The cleaning process of nanoparticles was performed both with hexane (Sigma-Aldrich, St. Louis, MO, USA) and deionized water (COCIBA, Querétaro, Mexico) in ultrasound baths and this process was repeated for several times.

### 2.2. Characterization

All the samples were characterized using the synthesized colloidal solution. Copper grids with carbon film were prepared with a drop of the colloidal solution for electron microscopy images. The samples were analyzed using aberration-corrected scanning transmission electron microscopy (STEM) with a Jeol ARM200F (200 keV) FEG-TEM/STEM microscope (Tokyo, Japan) equipped with a CEOS Cs-corrector (Heidelberg, Germany) on the illumination system. The optical parameters of the electron microscope are: spherical aberration coefficient = 1 micron, coma = 75.85 nm, two-fold astigmatism = 1.722 nm, three-fold astigmatism = 33.75 nm and star aberration = 0.515 nm. High-angle annular dark field (HAADF)-STEM images were obtained. The probe current used in STEM mode was 23.2 pA using a condenser lens aperture size of 40 microns. The HAADF images were registered using a camera length of 80 mm and a collection angle of 50–180 mrad. Average particle size was calculated by averaging 250 particles from the HAADF-STEM images using ImageJ software (v. 1.5, Analyze Particles module) developed at the National Institutes of Health (NIH). Energy-dispersive X-Ray spectroscopy (EDS, Oxford Instruments, Abingdon, UK) was performed by means of the Oxford system. Parallel beam X-ray diffraction (XRD) analysis of the bimetallic nanoparticles was carried out on a Rigaku Ultima IV X-ray diffractometer (Tokyo, Japan) equipped with a Cu K*α* source (*λ* = 0.154 nm) at 40 keV and 30 mA. The 2*θ* angular region between 30° and 90° is recorded at a scan rate of 0.05°/step with a speed of 2°/min.

### 2.3. Image Simulations

HAADF-STEM image simulations have been performed using the QSTEM software package (v. 2.22) [20], which uses the multislice algorithm based on the physical optics theory of Cowley and Moodie [21]. The QSTEM code is based on the methods described by E.J. Kirkland [22]. The parameters considered for the simulation correspond to the optical conditions of the electron microscope.

### 2.4. Molecular Dynamics Simulation

Molecular Dynamics (MD) simulations of three alloyed-bimetallic NPs with *N* = 923 atoms and cubo-octahedral structure were carried out. The alloys were randomly generated at different stoichiometric concentrations of Pd and Pt atoms: 2:1 (614 and 309 atoms), 1:1 (461 and 462 atoms) and 1:2 (309 and 614 atoms), respectively. All NPs were simulated using the Large-scale Atomic/Molecular Massively Parallel Simulator (LAMMPS, v. 2), an open source code for classical molecular dynamics simulations [23]. To describe the interatomic interactions in nanoalloys the many-body Gupta potential [24], as formulated by Cleri and Rosato [25] was used. Gupta potential is one of the most useful potentials in studying face-centered-cubic (FCC) metals and their alloys. To study the morphological and structural changes, thermal stability as well as surface segregation trends, all monometallic NPs and nanoalloys were heated in a temperature range from 300 to 1600 K with an increment step of 5 K in the vacuum atmosphere, using the canonical ensemble NVT and the application of a Nose-Hoover thermostat [26] for maintaining the constant temperature condition with a time step of 0.001 ps, considering a production time of 1000 ps and over which the average of the different physical properties studied was carried out. For obtaining structures of minimal energy, the conjugate gradient method was performed before starting simulations. Newton’s equations of motion applied to each atomic system were integrated using the Verlet-velocity integration algorithm [27]. In addition, temperature-dependent bond order parameters and potential energy for each nanostructure were calculated and plotted. Furthermore, the cone algorithm [28] was used to compute the number of Pd and Pt atoms residing on the nanoparticle’s surface. Both for heating and cooling processes, the same research protocol was followed, that is, all the parameters used in the molecular dynamics simulations were kept on the same. For each system, simulations were replicated ten times and all the graphs shown throughout the work resulted from the averages made with these replicas.

## 3. Results and Discussion

**Figure 1:**
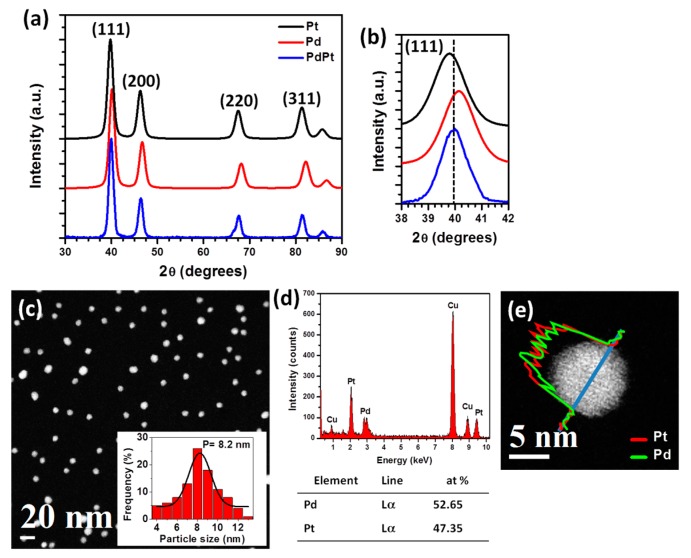
(**a**) X-ray diffraction (XRD) patterns of Pt, Pd and PdPt nanoparticles, (**b**) magnified zone of the (111) reflection, (**c**) low magnification high-angle annular dark-field scanning transmission electron microscopy (HAADF-STEM) image, (**d**) Energy-dispersive X-ray spectroscopy (EDS) spectrum of the bimetallic nanoparticles and (**e**) EDS line scan profile of a single bimetallic nanoparticle.

Parallel beam X-ray diffraction (XRD) measurements were carried out to determine the structure of the nanoparticles. Figure 1a shows the XRD patterns of the Pt, Pd and PdPt nanoparticles. Indexing XRD patterns correspond to the face-centered-cubic (FCC) lattice of Pt (JCPDF 04-0802), Pd (JCPDF 46-1043) and PdPt alloy (JCPDF 65-6418) structures. Lattice parameters of Pt and Pd are close, 0.3923 and 0.389 nm respectively, while the parameter of PdPt, 0.3896 nm, is between those two values, although nearer of the Pd-parameter. Consequently, the reflection positions of the PdPt planes are different from those of the pure Pt and Pd elements, but they are between both of these. The analysis of the position of the diffraction peaks can help or orient to identify the structure of the bimetallic nanoparticles. As can be observed in the Figure 1b, the position of the (111) Bragg reflection (or peak) of the PdPt is in 2*θ* = 39.98°; this diffraction angle is between those of Pt (39.8°) and Pd (40.15°), it indicates an average lattice parameter as usually expected for an alloyed structure, that would be the structure of PdPt. The properties of polycrystalline materials depend, among other things, on the crystallite size. In the case of nanoparticles, the crystallite size is associate with the average nanoparticle size [29]. The average crystalline domain size (t) of the bimetallic nanoparticles was calculated using the Scherrer equation [30]:

(1) $$t=\frac{K\lambda }{\beta cos\theta }$$

where *λ* is the X-ray wavelength in nanometers (nm), *β* is the full width at half maximum of the diffraction peak in radians, and *K* is a constant related to crystallite shape, normally taken as 0.9; the (111) diffraction peak of the XRD profile was used, leading to an average size of 8 nm. Figure 1c shows a low magnification high-angle annular dark field of scanning transmission electron microscopy (HAADF-STEM) image of the PdPt bimetallic nanoparticles. As can be observed in the image, the nanoparticles showed a particle size distribution almost homogeneous, with an average particle size of 8.2 nm (inset plot); this value is similar to the obtained by XRD. Average particle size was calculated by using ImageJ program (v. 1.5), which is a free package for image processing and manipulation, the average particle size was measured assuming a spherical shape of the nanoparticles. It is very important to mention that coalescence of the nanoparticles was not observed, also the image shows a homogeneous contrast of the nanoparticles giving a confirmation of the alloyed structure; while contrast variation within a single nanoparticle is not observed, which is a key point for core-shell structure identification [31]. Figure 1d shows the energy dispersive X-ray spectrum (EDS) acquired from the bimetallic nanoparticles showing both Pt and Pd peaks suggesting that both elements are present in the sample (Cu signal is from the copper grid). The atomic percentage of the Pd and Pt elements is about 52.65 at% and 47.35 at%, respectively. The compositional distribution of single bimetallic nanoparticle was obtained using EDS line scan profile. As described in Figure 1e, the line profile shows that the intensity of Pt and Pd is the same. This result suggests strong evidence for the formation of a bimetallic PtPd nanoparticle. 

**Figure 2:**
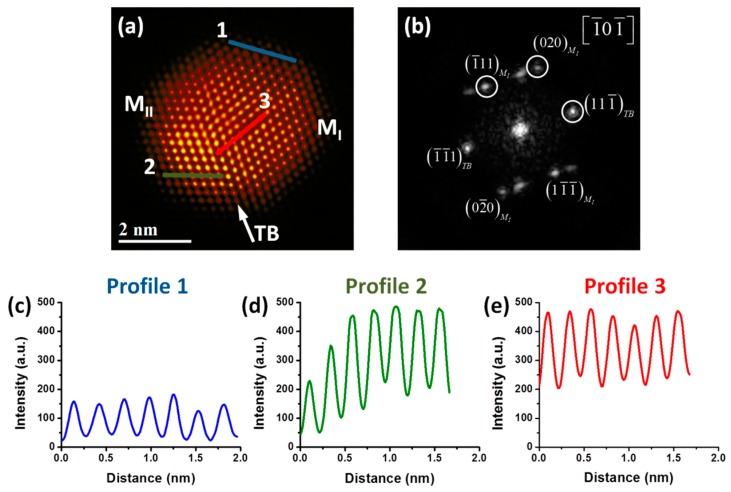
(**a**) High-angle annular dark-field scanning transmission electron microscopy (HAADF-STEM) image of a PdPt nanoparticle, (**b**) Fast Fourier transform (FFT) showing the twin boundary plane, and intensity profiles along (**c**) blue line, (**d**) green line and (**e**) red line, respectively.

As mentioned above, Pd, Pt and PdPt have almost similar lattice parameter. So that it is necessary a characterization technique that can give us more information, in this sense scanning transmission electron microscopy (STEM) is a technique that allows obtaining atomic information and, therefore, to understand the property-structure relationships. Figure 2a shows a high-resolution HAADF-STEM image of a PdPt bimetallic nanoparticle that displays two crystals (M_I_ and M_II_) separated by a single twin boundary (TB), which causes perturbation of the nanoparticle surface and it can be indexed as the [111] plane. Fast Fourier transform (FFT), seen in Figure 2b, shows a dual-spot diffraction characteristic of twinning, where d-spacings of M_I_ 0.2246, 0.2257 and 0.2018 nm were obtained, which correspond to (11-1), (1-1-1) and (020) crystalline planes respectively. Similarly, d-spacings of M_II_ 0.2311, 0.2257 and 0.2016 nm were obtained, which correspond to (11-1), (1-1-1) and (020) crystalline planes respectively. Therefore, the nanoparticle is viewed along the [-10-1] zone axis confirmed by the FFT. The presence of twining defects in the produced alloyed nanoparticles is also a key feature of the material, because they improve the catalytic activity in heterogeneous catalysis, and also tend to modify the facet types and proportions of surface atoms [32]. Also, in Figure 2a differences in contrast are clearly visible; an effect that is associated with the atomic number of the elements in the sample (Z contrast) in HAADF-STEM images [33]. Therefore, the strong brightness zones of the image correspond to the heaviest element (Pt) and the low brightness zones correspond to the lightest element (Pd). To visualize better this contrast, Apply_CLUT (color look-up table) script was used in the image [34]. As can be clearly observed from the figure, few regions are more intense than the others ones, indicating a smaller quantity of Pt atoms in the surface that Pd. The difference in intensity brightness is an indication of the presence of both Pd and Pt atoms at the surface. A study about the identification of the atoms in PdPt bimetallic alloy nanoparticles using HAADF-STEM images has been reported previously by our group [35]. This study showed a semi-quantitative method to analyze the atomic distribution of the elements, which is dependent of their atomic number. Intensity profiles were performed to analyze the intensity brightness of the different atomic columns, and it is important to mention that the graphics have the same intensity scale for a better comparison. Intensity profile 1, seen in Figure 2c, was measured along the M_I_ crystal of the nanoparticle. The intensity columns are uniform, indicating that the atomic columns are from the same type of atom, which according to the HAADF-STEM image could be associated with the Pd atoms. Intensity profile 2, shown in Figure 2d, was measured along the M_II_ crystal of the nanoparticle, which shows more diverse intensity columns than profile 1: the difference in intensity among the first and last columns is higher, indicating clearly that some columns are Pt rich compared to other ones, and that the Pt atoms are not uniform in the surface. This behavior is the result of Pt rich columns, which appear much brighter in the image, and with more intensity scale in the graphic. Intensity profile 3, shown in Figure 2e, was measured in the middle portion of the nanoparticle (M_I_ crystal). In this profile, the intensity columns are almost uniform and higher, indicating that in this zone there are mainly Pt rich columns. 

**Figure 3:**
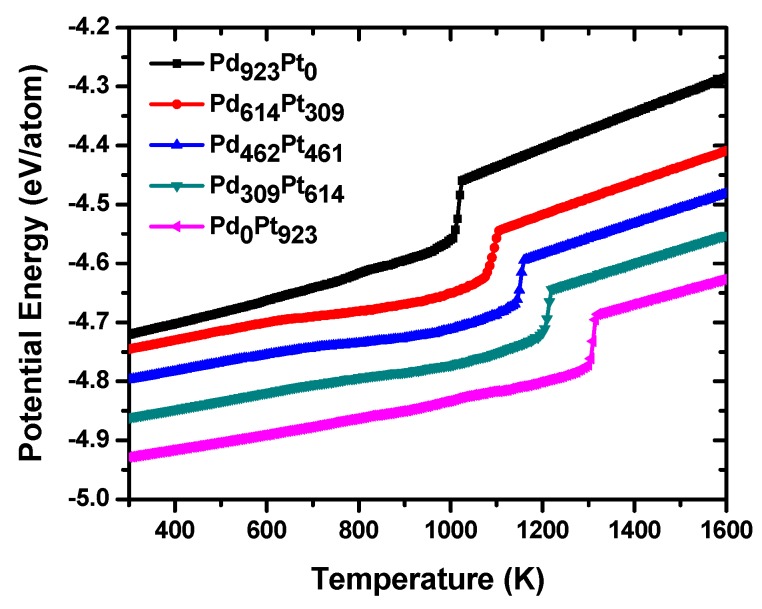
Temperature-dependent average potential energy of nanoparticles. In the graph, the values of the potential energy for each nanostructure were rescaled so that the distinctive features of each curve could be better appreciated.

The above results show that there is high amount of Pd atoms in the particle surface; however, to get more information about the surface atomic segregation phenomenon, molecular dynamics simulations were performed. As it is known, for investigating the melting behavior of a nanoparticle there are different criteria to identify the phase transition temperature from solid to liquid state. One of them is the variation of the potential energy with temperature, which has been used for calculating the melting points of each nanoparticle. Figure 3 shows the dependence of the average potential energy versus temperature for the PdPt (2:1), PdPt (1:1) and PdPt (1:2) alloyed bimetallic and Pd_923_ and Pt_923_ monometallic NPs during the heating process from 300 to 1600 K. Two important features stand out in the figure: both the thermal stability and the melting points of the nanoalloys increase as the Pt content increases and on the other hand, the three nanoalloys are more thermally stable than the Pd nanoparticles, but have a less thermal stability than Pt_923_. As the heating process develops, all nanoparticles exhibit a line arity period in their potential energy curves which is different for each structure and depends significantly on the atomic composition. In the case of monometallic systems, this period of linearity is somewhat longer than in the case of nanoalloys and has slight oscillations before the occurrence of the solid-liquid phase transition. The fact that the linear stage in Pt is larger than in Pd nanoparticle can be explained by the fact that the first nanostructure is thermally more stable than the second one as it is known from previous reports [19,36] and consequently that a greater amount of thermal energy is required to melt the particle. As will be seen later, these small oscillations in the curves of potential energy obey to slight morphological and structural changes in the particles, which do not affect much to their original structure. As expected, in the case of bimetallic systems this period of linearity increases as the content of platinum increases in the nanoalloys because the latter element is more stable than palladium; therefore, of all nano-alloys, those that are richest in platinum are the most thermally stable. In these periods of linearity both nanoalloys undergo almost imperceptible structural transformations, so that their morphologies are almost identical to those of the original structures at 300 K. After this stage, an important period of non-linearity occurs for each of the nanoalloys; in these periods the slopes of curves are changing continuously until a sudden increase in the potential energy occurs. The melting point of each nanoparticle can be identified from its corresponding caloric curve; that is, the temperature value at which a sudden increase in potential energy occurs. From the figure, it can be seen that for Pt_923_, the melting temperature (1304 K) is higher than that of Pd_923_ nanoparticle (1010 K), and these temperature values are less than the corresponding bulk melting points [37,38]. The period of nonlinearity is longer in the nanoalloys that have a higher content of palladium and plays a very important role during the melting process because in it, the Pd atoms begin to diffuse towards the surface of the particles. In the same way, and as it will be corroborated later, both nanoalloys do not undergo significant structural and morphological changes until temperatures close to the melting point are reached; here, nanoparticles begin to undergo strong structural transformations, beginning a superficial premelting phenomenon, and the crystalline lattice begins to be destroyed. Because the phenomenon of melting starts from the surface and evolves into the inner of particles, those structures whose surface is richer in Pd atoms melt at lower temperatures since the latter has a surface energy lower than that of Pt. This fact explains why those structures that are richer in Pt atoms show a shift to the right at their melting points, getting closer and closer to the melting temperature of the pure Pt nanoparticle. During the stages of surface premelting and melting transition, an interesting phenomenon occurs in all nano-alloys: Pt and Pd atoms are exchanged and a considerable number of atoms are in a liquid-like state, i.e., there is a significant contribution of the atomic anharmonic motion. Temperature values that are very close to the melting transition temperatures can be considered to be like the limit points for the stability of crystalline matrix in each nanostructure and for temperatures above these values, the last begins to be destroyed. While on the one hand, the Pd atoms are migrating towards the surface of particles, on the other, the Pt ones diffuse towards their inner cores taking place the phenomenon of intermixing. Once the melting transition is completed, all the particles are in liquid state and remain so until the completion of the heating process at 1600 K.

For the case of bimetallic NPs, melting points for both nanoalloys are the following: 1087 K for PdPt (2:1), 1140 K for PdPt (1:1), and 1200 K for PdPt (1:2), and each one is included between the values corresponding to the monometallic systems. However, the significant differences observed in melting points could be explained not only by considering the number of atoms of each species in nanoalloys, but by the way in which the Pd and Pt atoms are distributed in the clusters.

**Figure 4:**
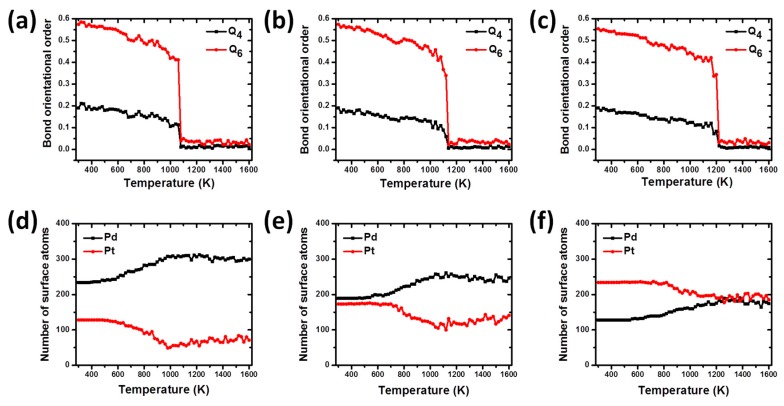
(**a**–**c**) Temperature-dependent bond order parameter, and (**d**–**f**) number of surface atoms, for the for bimetallic nanoparticles PdPt (2:1), PdPt (1:1), and PdPt (1:2), respectively.

Figure 4 shows both the temperature-dependent global bond order parameters *Q*_4_ and *Q*_6_, and the number of Pd and Pt surface atoms for the Pd-Pt-alloyed bimetallic nanoparticles. All the values shown in plots were theoretically calculated for each nanostructure upon heating process. The calculation of global bond order parameter is a very useful method to investigate the structural evolution of nanoparticles [28]. As it is known, there are several criteria to identify the local and global orientation symmetries of clusters. One of these criteria is the bond order parameter method [39], which is very useful to analyze and get information about the cluster structure as well as to distinguish between atoms in solid (close packed) and liquid states originated during melting processes. It is not only a sensitive indicator of the melting transition, but also a measure for identifying the structural change of nanoparticles upon heating and freezing processes. The global order parameter *Q*_l_, is defined as:

(2) $$Q_{l}={\left(\frac{4\pi }{2l+1}\overset{l}{\underset{m=-l}{\sum }}{\left|{\bar{Q}}_{lm}\right|}^{2}\right)}^{1/2}$$

where:

(3) $${\bar{Q}}_{lm}=\frac{\sum_{i=1}^{N}N_{\mathrm{nb}}\left(i\right)q_{lm}\left(i\right)}{\sum_{i=1}^{N}N_{\mathrm{nb}}\left(i\right)}$$

*N* is the total number of atoms in the particle, *N*_nb_(*i*) is the number of first neighbors for the *i*-th atom, and *q*_6*m*_(*i*) measures the local order around the *i*-th atom considering the average of the spherical harmonics *Y*_6*m*_ of the bonds with the *N*_nb_(*i*) neighbors:

(4) $$q_{6m}\left(i\right)=\frac{\sum_{j=1}^{N_{\mathrm{nb}}}Y_{6m}\left(r_{ij}\right)}{N_{\mathrm{nb}}\left(i\right)}$$

Before starting the relaxation and simulation of nanoalloys, the values for the bond order parameters *Q*_4_ and *Q*_6_ were calculated for the designed models, obtaining a good agreement with the values reported for ideal structures [28]. As can be seen from the Figure 4, the bond order parameter curves for the bimetallic nanoparticles show a slight decrease from 300 to 1060 K for PdPt (2:1), as seen in Figure 4a, 1080 K for PdPt (1:1), shown in Figure 4b, and 1160 K, as seen in Figure 4c, for the PdPt (1:2) nanoparticles. These results show that in this temperature range, the structures do not show significant structural changes and the nanoparticles are experiencing contractions and expansions around their centers of mass. At this stage, slight oscillations are observed in each of the curves and generally they can be attributed to the fact that all atoms are experiencing chaotic and disordered movements due to excess thermal energy absorbed by the systems. However, after the mentioned temperatures, a rapid and sudden drop in these values occurs until the phase transition temperature from solid to liquid is reached. During this solid-liquid transition particles undergo strong changes in their morphology and are in an amorphous state. The last temperature values are in good agreement to those observed in the caloric curves, where they were identified as the melting points for both nanoparticles.

One of the methodologies used to investigate the atomic segregation on the surface of Pd-Pt alloyed nanoparticles is the “cone” algorithm [28] which is based on identifying the number of atoms on the particle surface from their geometric positions. The dependence of the number of Pd and Pt atoms on the surface particles as a function of temperature can be observed in Figure 4. As can be seen, Figure 4d shows that for the nanoalloy PdPt (2:1) there is a greater amount of Pd atoms on the surface in relation to those of Pt at the beginning of heating process. These Pd atoms remain all the time on the particle surface, while those in the interior keep on migrating to the surface during the premelting stage, which is longer than in the case of the alloy PdPt (1:1) and PdPt (1:2). It is known that the melting phenomenon starts in the surface and evolves towards the interior of the particle until it is completely melted. At this point, the structure begins to undergo considerable structural transformations and the phenomenon of atomic diffusion begins to occur, showing a clear tendency of the Pd atoms to segregate on the particle surface and in addition, of Pt atoms to diffuse towards its inner core.

A similar behavior is observed for nanoalloy PdPt (1:1), shown in Figure 4e, in the variation of the number of the atomic species on the particle surface: the Pd atoms prefer to remain on the particle surface throughout the heating process. In this case, the amount of surface Pd atoms at the end of the heating process is lower than that observed in the alloy PdPt (2:1) (246 and 300, respectively); and this could be explained by the fact that the last nanostructure has the highest number of Pd atoms and this metallic species prefers to diffuse towards the surface of nanoparticles.

On the other hand, for the nanoalloy PdPt (1:2), seen in Figure 4f, the number of Pd surface atoms on the structure is smaller than that of Pt ones, in a temperature range from 300 to 1200 K. In the temperature range from 300 to 800 K, the number of Pd and Pt atoms on the particle surface remains approximately constant at the values of 143 for Pd and 232 for Pt. In this temperature interval, the nanoparticle does not exhibit significant structural changes, retaining many of the distinctive features of the original structure. However, after 800 K and before 1200 K, an increase in the amount of Pd atoms on the surface can be observed as well as a decrease in the number of Pt atoms, until both atomic species have almost the same amount of atoms. The above results show that as temperature increases, the Pd atoms show a tendency to migrate towards the particle surface in all Pd-Pt bimetallic NPs. This behavior has also been observed in others Pt-based bimetallic systems where is necessary to anneal the sample at high temperatures to promote surface segregation [1]. In addition, both Pt and Pd are totally miscible and their bulk melting temperature is 2045 K and 1827 K, respectively, so according to the segregation rules reported previously by G. Guisbiers et al., the segregated element is the one with the lowest surface energy, therefore Pd goes to the surface due to Pd having a lower surface energy compared to Pt [40]. This evidence shows a trend of Pd atoms to segregate on the surface of nanoparticles and to remain there even after the completion of the heating process in correspondence with the results published in previous works [19,41], i.e., C. Fernández-Navarro et al. [42] studied the dynamics of Pd-Pt nanoparticles in a process of heating until reaching the melting condition, and compared the melting process in an unsupported particle against that of a particle supported on a graphite substrate. Their results exhibited a local rearrangement of metal atoms near the carbon substrate in such a way that the metal lattice had a better match with the carbon substrate, and that the Pd atoms migrated to preferentially occupy the region in direct contact with the carbon substrate.

**Figure 5:**
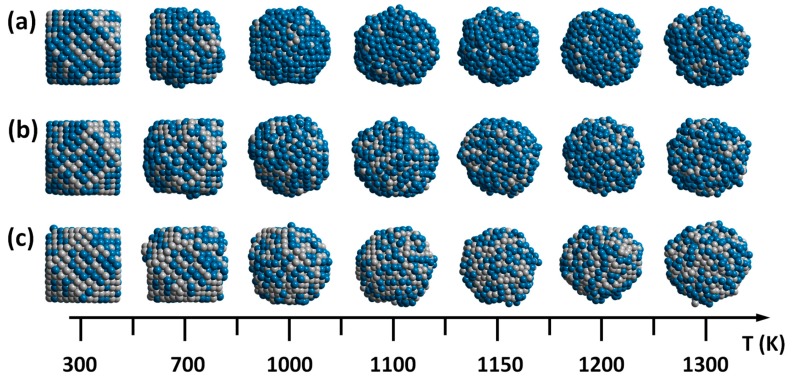
Final configurations after 1000 ps MD run of the nanoparticles at various temperatures for: (**a**) PdPt (2:1), (**b**) PdPt (1:1), and (**c**) PdPt (1:2) nanoparticles, respectively (Pd = blue and Pt = grey).

Figure 5a–c represents the final configurations after 1000 ps MD run of the PdPt (2:1), PdPt (1:1), and PdPt (1:2) nanoparticles at various temperatures, respectively. The bimetallic nanoparticles retain their initial cubo-octahedral structure at 300 K, which is bounded by {100} and {111} facets. However, it is clear from the figures that the evolution in the structure of the particles depends on the concentration of the components and the temperature. As mentioned previously, the PdPt (2:1) has a lower melting point than those of PdPt (1:1) and PdPt (1:2), which can be clearly observed from the configurations of Figure 5, where the {100} and {111} facets of the nanoparticle remain almost without significant deformations between 300 K and a temperature below 1000 K; in addition this configuration tends to form a core-shell structure, due to the fact that Pd is enriched in the shell and therefore reduced in the core. The configuration PdPt (1:1), the {100} and {111} facets remain almost without deformation until 1100 K. In this case, less Pd atoms enrich the surface of these nanoparticles. In the configuration PdPt (1:2), the {100} and {111} facets remain almost without deformation until 1200 K and an alloy structure is formed in the surface of the nanoparticle with more Pt atoms. At the end of the heating process the nanoparticles have an amorphous structure, where the atoms can pack as close as possible, in order to maximize the cohesive energy.

**Figure 6:**
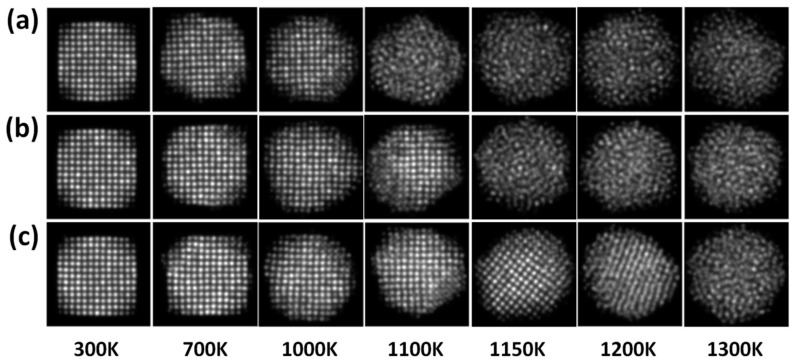
Simulated High-angle annular dark-field scanning transmission electron microscopy (HAADF-STEM) images at various temperatures for the structures: (**a**) PdPt (2:1), (**b**) PdPt (1:1) and (**c**)PdPt (1:2), respectively.

Figure 6a–c shows the simulated HAADF-STEM images of PdPt (2:1), PdPt (1:1), and PdPt (1:2) nanoparticles obtained at the same temperatures used for the final configurations as shown in Figure 5. The simulated images were acquired using the optical parameters of the electron microscope. The aim of simulated HAADF-STEM images is to understand the effect of the contrast associate to the Pd and Pt elements (Z-contrast) and to analyze in detail the structure in the image. The images were recorded using the same intensity scale, with the purpose of comparing the intensity contrast among them. As can be observed from the simulated images, in all cases the intensity (brightness) contrast significantly decreases as temperature increases. As is known, the contrast in high resolution electron microscopy images is more sensitive to the crystallographic nature of specimens [43], that is, in a crystalline specimen the intensity of the scattered electron beams has a maximum at certain specific angles due to the interplanar spacings. However, in an amorphous specimen, all the atoms are randomly distributed through its volume, so they are not really arranged inside it, but there are a few interatomic spacings that tend to occur in the amorphous structure. Therefore, a diffuse contrast is observed in the images [44].

**Figure 7:**
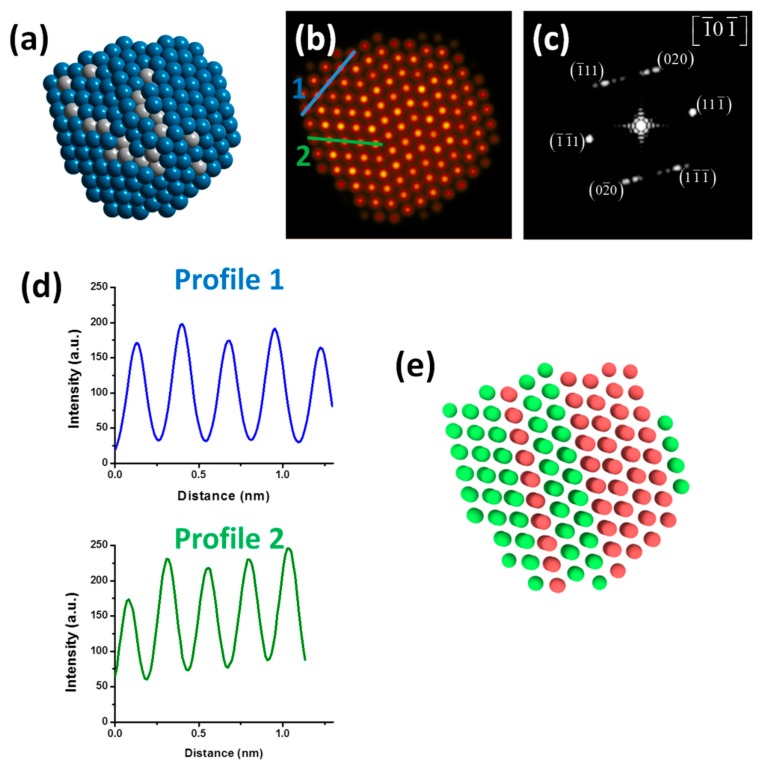
(**a**) Model of the PdPt (1:1) nanoparticle after the cooling process (Pd = blue and Pt = grey), (**b**) Simulated high-angle annular dark-field scanning transmission electron microscopy (HAADF-STEM) image of the PdPt (1:1) nanoparticle, (**c**) Fast Fourier transform (FFT) of the simulated HAADF-STEM image, (**d**) intensity profiles along two directions and (**e**) Atoms colored according to their crystal stacking structure calculated using a common neighbor analysis (green = FCC and red = HCP).

After the completion of cooling processes, significant surface reconstructions can be made for the final nanostructures at 300 K given that from the melted nanostructures a great diversity of structures could occur after the liquid to solid phase transition. Something important that must be pointed out is that after carrying out the cooling processes, of the total of simulated replicas, seven of them fell into FCC twinned-type bimetallic NPs while the rest ended in decahedral nanostructures. Figure 7 shows the results obtained at 300 K after the completion of the cooling process (crystallization) for the PdPt (1:1) nanoparticle. In this work, only the results corresponding to the PdPt composition (1:1) are shown to have a greater similarity with the experimental results and consequently, a better comparison criterion. Figure 7a shows the model of the PdPt (1:1) alloyed bimetallic nanoparticle. In the image, most of Pd atoms are located on the particle surface, and it was constructed from the calculation of the average of atomic positions of each metallic species at 300 K (ten samples were considered, as mentioned above). Figure 7b shows the simulated HAADF-STEM image of model; to better visualize the contrast of the image, the Apply_CLUT (color look-up table) script was used. The simulated image displays some crystalline defect (twin boundary), which causes perturbation in the nanoparticle. FFT of the simulated image, as seen in Figure 7c, shows a dual-spot diffraction characteristic of twinning and the nanoparticle is viewed along the [-10-1] zone axis. Figure 7d shows the intensity profiles measurements over two directions; the intensity profiles show diverse intensities; this behavior is characteristic of Pd rich columns and Pt rich columns. Finally, Figure 7e shows the atoms colored after a common neighbor analysis (CNA) using OVITO program (v. 2.9) [45]; which is used to determine the local crystalline order of the bimetallic nanoparticles, to possibly identify dislocations, stacking faults, deformation twinning, and structural evolution [46]. From the figure, green and red coloring represent atoms with an FCC and HCP (twinning) stacking, respectively. As can be observed there is good agreement between the experimental and the simulation results.

## 4. Conclusions

PdPt bimetallic nanoparticles with alloy structure and homogeneous particle size of 8.2 nm were synthesized by the polyol method. The bimetallic nanoparticles were characterized by XRD, EDS and HAADF-STEM, which is dependent of the atomic number of elements; each technique showed that the nanoparticles have an alloy-like structure. The intensity profiles of HAADF-STEM images show that there are atomic columns with Pd content higher than that of Pt. Temperature-dependent average potential energy, bond order parameters, and number of surface atoms for PdPt nanoparticles with 923 atoms, have been simulated by molecular dynamics under the canonical ensemble NVT and using the Gupta potential. From a thermodynamic point of view, the results obtained show that nanoalloys PdPt (2:1), PdPt (1:1) and PdPt (1:2) exhibited thermal stabilities higher than Pd but lower than that of Pt monometallic nanoparticles. The obtained results showed that melting points of nanoalloys underwent a shift towards higher temperature values as the Pt content increases in the particles, and in addition, an increase in their thermal stability. The global bond order parameters *Q*_4_ and *Q*_6_ showed that all nanoalloys experienced significant structural changes with the temperature increasing. The values calculated for these parameters are evidence that as the heating process develops, all nanoparticles undergo structural transformations, ranging from FCC structures to amorphous structures (structures in liquid state). On the other hand, for analyzing the surface segregation phenomenon, the number of Pd and Pt surface atoms was calculated for bimetallic nanoparticles. The criterion clearly predicts both a trend of the Pd atoms to segregate on the nanoparticle surface as temperature increases, and to mostly to stay on it. The common neighbor analysis was performed after the completion of cooling process (300 K), to determine the local crystalline order of the PdPt (1:1) bimetallic nanoparticle showing both zones with FCC and HCP (twinning) stacking. We can conclude that the results obtained from both the experimental and the simulated experiments are in agreement.
